# Enhanced Recovery after Surgery (ERAS) for Minimally Invasive Gynecologic Oncology Surgery: A Review

**DOI:** 10.3390/curroncol30100677

**Published:** 2023-10-22

**Authors:** Christa Aubrey, Gregg Nelson

**Affiliations:** 1 Department of Obstetrics & Gynecology, Faculty of Medicine and Dentistry, University of Alberta, Edmonton, AB T6G 1Z2, Canada; 2 Department of Obstetrics & Gynecology, Cumming School of Medicine, University of Calgary, Calgary, AB T2N 4N2, Canada; gregg.nelson@albertahealthservices.ca

**Keywords:** enhanced recovery after surgery, ERAS, minimally invasive surgery, MIS, perioperative care

## Abstract

Enhanced recovery after surgery (ERAS) has established benefits in open gynecologic oncology surgery. However, the benefits for gynecologic oncology patients undergoing minimally invasive surgery (MIS) are less well defined. We conducted a review of this topic after a comprehensive search of the peer-reviewed literature using MEDLINE and PubMed databases. Our search yielded 25 articles, 14 of which were original research articles, in 10 distinct patient cohorts describing ERAS in minimally invasive gynecologic oncology surgery. Major benefits of ERAS in MIS included: decreased length of stay and increased rates of same-day discharge, cost-savings, decreased opioid use, and increased patient satisfaction. ERAS in minimally invasive gynecologic oncology surgery is an area of great promise for both patients and the healthcare system.

## 1. Introduction

Enhanced recovery after surgery (ERAS) is now established as the standard of care across the majority of surgical disciplines [1]. ERAS represents a collaborative, bundled intervention for perioperative care in which many interventions alone translate to meaningful improvements but implemented together contribute synergistically to meaningful gains. These consist of preoperative medical optimization, standardized intraoperative anesthetic, perioperative fluid management aimed at euvolemia, postoperative multimodal analgesia, thromboprophylaxis, and early return to function including ambulation, catheter removal, and nutrition [2]. Initially, ERAS gained popularity in colorectal surgery in the early 2000s and followed suit in 2016 with the development of ERAS guidelines in gynecologic oncology, which were subsequently updated in 2019 and 2023 [2,3,4]. During this time period, there has been increasing evidence demonstrating the benefits of ERAS in open gynecologic oncology surgery primarily, including decreased length of stay, complications, and cost, balanced by no increase in morbidity [5].

There has been some skepticism regarding the additional benefit of ERAS when applied to minimally invasive surgery (MIS). However, there is still much room for improvement in outcomes in MIS gynecologic surgery, with the rates of same-day discharge (SDD) demonstrated to be only 30%, and without targeted interventions, these rates are only projected to marginally increase, despite accepted SDD safety and feasibility [6]. Another issue, particularly relevant to the North American context, is the opioid crisis, which has been driven to some degree by the inappropriate prescribing of opioids after surgery. This realization led to a call to action from the United States Surgeon General in 2016 for safer opioid prescribing practices [7]. The recent 2023 update of the gynecologic oncology guidelines specifically addresses implementation challenges including creating successful SDD programs through collaboration, education, patient selection, and ERAS perioperative principles, including appropriate postoperative opioid prescribing [4,8].

The aim of this review was to summarize the impacts of ERAS in patients undergoing minimally invasive gynecologic oncology surgery. 

## 2. Materials and Methods

A comprehensive search of the peer-reviewed literature was conducted using MEDLINE and PubMed databases in September 2023. Search terms included a combination of “ERAS, or Enhanced Recovery after Surgery”, “MIS, or Minimally invasive surgery”, and “Gynecologic Oncology”. References of relevant articles were also screened. Full-text review of all results was undertaken and included studies consisting of peer-reviewed publications that met the following inclusion criteria: (1) English language, (2) specific reference to ERAS, MIS, and gynecologic oncology. Studies that were not limited to MIS, or gynecologic oncology were included if they had a subset analysis that detailed these groups separate from overall more inclusive groupings. There were no limitations on dates of publication. Review articles and commentaries were taken into consideration, but original research studies were only eligible for inclusion in the summary. Exclusion criteria included: (1) non-English language, (2) studies that did not include populations specific to gynecologic oncology, or MIS.

## 3. Results

The search yielded 25 articles, 14 of which were original research articles, in 10 distinct patient cohorts describing ERAS in minimally invasive gynecologic oncology surgery. Table 1 summarizes these studies and their findings. Eleven of the 14 articles dealt exclusively with MIS gynecologic oncology surgery, 1/14 included both open and MIS procedures, and 1/14 included both gynecologic oncology and non-gynecologic oncology MIS surgeries (both provided distinction of the ERAS MIS gynecologic oncology surgeries within the cohorts). The majority of studies included both laparoscopic and robotic surgical approaches, and a variety of different gynecologic cancer diagnoses (endometrial, ovarian, cervical, and “other”). Most studies did not detail the specific surgical procedures included. There were four distinct outcomes discussed within these articles with regards to the benefit of ERAS in MIS gynecologic oncology surgery: (1) decreased length of stay, and increased rates of same-day discharge, (2) cost savings, (3) decreased opioid use in the perioperative and postoperative period, (4) patient satisfaction. 

### 3.1. Decrease Length of Stay, and Increased Rates of Same-Day Discharge

Decreased length of stay in post-ERAS implementation in MIS gynecologic oncology surgery was described initially by Chapman et al., where a 2:1 matching of historical controls to 55 post-ERAS MIS (laparoscopic or robotic) gynecologic oncology surgery patients was described. In this study, controls were matched to age and surgery type, with no difference in demographic or clinical factors. They found in the ERAS cohort there was a greater likelihood of being discharged on the first day after surgery (91% vs. 60%, *p* < 0.001), and hospital stay was a median of 4 h less in the ERAS MIS group [13]. In a prospective observational study of gynecologic oncology surgery, ERAS with MIS was shown to be an independent factor for early discharge (OR 0.02, 95% CI 0–0.07, *p* < 0.001) [20]. In a retrospective pre/post study (n = 86, with 65 historical controls), ERAS implementation in MIS gynecologic oncology surgery, was associated with a reduction in length of stay from 3.2 days to 0.9 days (*p* < 0.0001) [14]. Subsequently, a number of studies have reported similar outcomes to enhance SDD rates with ERAS implementation in MIS gynecologic oncology surgery patients. Brancazio et al., in a pre/post-ERAS MIS implementation study of 800 patients, 351 of whom were in the post-intervention arm, found that the standardized ERAS protocol alone increased the rates of SDD from 39.3% to 48.9% [9].

The safety and feasibility of SDD in ERAS MIS gynecologic oncology surgery was investigated in a retrospective cohort study by Wield et al., where 1124 patients were evaluated. SDD in this study was 69%, and safety outcomes of 30-day postoperative complications, emergency department visits, and readmission rates were reported, with 8.7% overall complications in the cohort, and among the SDD patients, there were significantly fewer complications [12]. They also described the clinicodemographic and operative factors associated with an increased likelihood of overnight admission and found that older age, distance from the hospital, longer procedure, complications, later start time, radical hysterectomy, mini-laparotomy, adhesiolysis, CAD, CVD, VTE, DM, and neurologic disorders were all associated [12]. To further bolster SDD, a quality improvement initiative aimed at improving rates of SDD in MIS gynecologic oncology surgery was described by Kim et al. In this study, the ERAS MIS cohort consisted of 102 cases, compared with 100 historical controls. Through a dedicated QI initiative, SDD rates were improved further from 29 to 75% post-intervention (*p* > 0.001) [17]. In their paper focusing on the specific population of ERAS MIS robotic endometrial cancer surgery, Mateshaytis et al. described a quality improvement initiative using a time series design with process and balancing measures [15]. Through the ERAS MIS bundled initiative they focused on improving patient education, early removal of the Foley catheter in the operating room, and default ordering of SDD. The rate of SDD improved from a baseline of 29.4% prior to the intervention to 78.3% post-intervention [15].

### 3.2. Cost Savings

Correlated with decreased length of stay related to ERAS implementation, two studies quantified the cost-saving benefits of ERAS MIS in gynecologic oncology surgery. Chapman et al. estimated costs before and after ERAS MIS implementation using a cost database^9^. Direct and indirect costs were calculated and reported relative to 2014 USD. Total hospital costs after ERAS implementation were estimated to be on average USD 1810 less per patient (12%), which was largely due to room costs [13]. After their quality improvement program to improve rates of SDD after MIS gynecologic oncology surgery, Mitric et al. estimated the economic impact of these cost savings [18]. Using micro-costing analysis of direct costs both perioperatively and postoperatively, the post-intervention cost savings were estimated to be CAD 1130 per patient which was due mostly to operating room cost savings and post-operative stay savings [18].

### 3.3. Decreased Opioid Use

**Figure 1:**
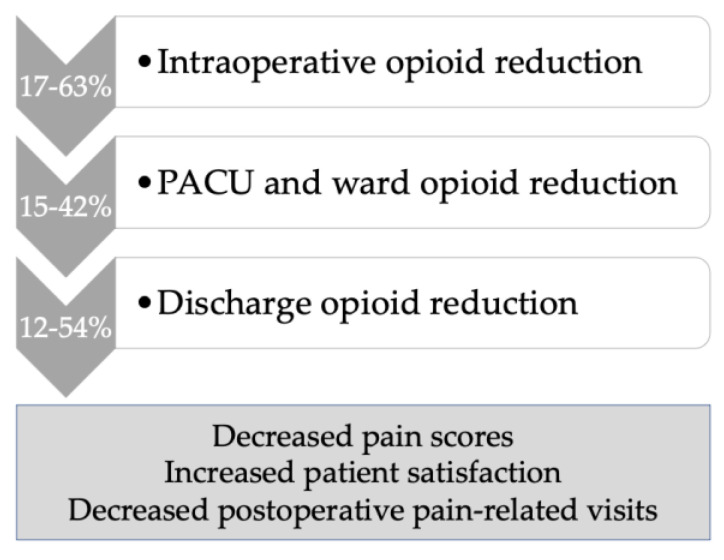
Opioid reduction within an ERAS MIS program in gynecologic oncology, using multimodal non-opioid analgesia.

There were five studies that reported on decreased opioid use in relation to ERAS MIS gynecologic oncology surgery. Within the ERAS bundled intervention that utilizes multimodal non-opioid analgesia, Chapman et al. demonstrated a 30% reduction in opioid use (31 mg vs. 44 mg IV morphine equivalents, *p* < 0.01) and despite this, also reported lower postoperative pain scores of 2.6 vs. 3.12 (*p* = 0.03) [13]. In a retrospective pre/post study of clinical outcomes following ERAS implementation for major gynecologic surgery, Modesitt et al. described an ERAS “light” protocol for MIS surgery that showed decreased opioid use in both the intraoperative and postoperative settings [21]. Similarly, in 126 ERAS MIS cases compared to 99 historical controls, fewer oral morphine equivalents (OME) were used in the ERAS MIS cohort both intraoperatively (10.43 OME fewer, *p* < 0.001), and postoperatively (10.97 OME fewer, *p* = 0.019) [22]. In this study, pain scores were also 0.56 points lower in the ERAS MIS cohort (*p* = 0.013) [22]. Lehman et al., in a cohort study of 351 ERAS MIS patients matched to 449 historical controls, less opioid use in ERAS MIS was found intraoperatively, with a nonsignificant trend to less opioids in the recovery room [10]. Levytska and colleagues, in a separate study based on the previous cohort, showed that although less opioid was prescribed, this did not lead to increased clinic burden but rather that postoperative phone calls and unscheduled visits for pain were reduced in the ERAS MIS cohort [11]. After the implementation of an opioid-restrictive program following ERAS MIS gynecologic oncology surgery, Kim et al. found that 75% of patients used less than 10 median morphine milligram equivalents, and 54% required no opioids postoperatively. Postoperatively, the number of opioid tablets prescribed was 5 vs. 10 tablets (*p* < 0.01) with no increase in opioid prescription refills [19]. This program consisted of patient and provider education, standardized intraoperative multimodal non-opioid analgesia, and standardized postoperative prescribing utilizing multimodal analgesia order sets. Postoperatively, patients received early education and telephone follow-up [19]. Figure 1 summarizes the benefits of an ERAS MIS program utilizing multimodal analgesia in gynecologic oncology with regard to opioid reduction in the various domains of patient care based on the studies presented above.

### 3.4. Patient Satisfaction

In a dedicated study to evaluate patient satisfaction with an ERAS MIS program in gynecologic oncology surgery, the EVAN-G validated questionnaire for perioperative patient satisfaction was reported in 92 patients and demonstrated that the majority of patients were either very (60.8%) or quite (32.6%) satisfied with the quality of care received; the general overall satisfaction was 81.9 (0–100 scale of satisfaction) [16]. Patient satisfaction was also demonstrated in the above-described study by Modesitt et al. in the domains of pain control, information, and perception of staff teamwork [21]. Patient satisfaction can also be extrapolated from the previously described studies which demonstrated lower pain scores [22], and calls/unscheduled visits for pain [11].

## 4. Discussion

This review demonstrates the benefits of ERAS in MIS gynecologic oncology surgery specifically decreased length of hospital stay, and increased rates of same-day discharge, without an increase in morbidity or readmission. Moreover, ERAS in MIS is associated with cost savings, together with a robust decrease in opioid use perioperatively and postoperatively without compromising reported pain scores and follow-up burden. Finally, our review found that ERAS implementation was associated with patient satisfaction. 

Same-day discharge has become the standard recommendation for MIS gynecologic surgeries. Even in an oncology patient population that differs from the general gynecologic population with respect to increased age, comorbidities, and surgical complexity, SDD has been deemed to be safe [23]. Despite this, there remains skepticism regarding the additional benefits of ERAS over and above that of MIS. However, Luchristt et al. conducted a National Surgical Quality Improvement Program (NSQIP) database analysis of the SDD rates from 2011–2019 and found that SDD rates were only 30% [6]. They predicted SDD rates to improve only to 48.5% by 2029 unless focused SDD improvement measures were instituted [6]. In our review of the literature, we found that an ERAS MIS protocol in gynecologic oncology improved the rates of SDD from a baseline of approximately 30% to 50–70%, depending on the extent of measures in place (patient education, standardized orders, prioritizing surgery earlier in the day, early Foley removal, reduction of opioids, and prophylactic anti-emetics) [9,15,17]. There remain barriers to SDD, and surgeon preference has been shown to be a major contributor, which is why it is imperative to develop robust eligibility criteria, based on safety data to determine those who are most able likely to benefit [4,9]. Exclusion criteria for SDD have included: comorbid conditions, patient age, home supports, distance from hospital, surgical factors, and increased BMI [17], although many of these exclusion factors may represent opportunities for further improvement in patient eligibility as the majority are not evidence-based. 

The economic impact of ERAS on the healthcare system is widely established [24]. In a recent systematic review and meta-analysis of the outcomes of ERAS in gynecologic oncology, the mean cost savings associated with ERAS care was estimated to be USD 2129 per patient [5]. In our review, cost savings were found to be between USD 1810 [13], and CAD 1129 [18] per patient. The economic benefits from ERAS are largely driven by reductions in length of stay, and within the MIS ERAS gynecologic oncology context, further benefits will depend on our discipline’s ability to continue to iterate towards improvements in SDD rates. 

Reduction in opioid prescribing practices has been a mainstay of ERAS programs. In gynecologic surgery, over-prescribing of opioids remains an issue, with studies demonstrating over 60% of patients receive postoperative prescriptions for opioids even after minor procedures [11]. Numerous studies within this review have demonstrated successful gains in perioperative opioid use [10,13,21,22] and postoperative opioid prescribing practices [11,19,22]. There is growing awareness of the implications of opioid over-prescribing as related to the opioid crisis, with 6% of new persistent opioid use attributed to post-surgical exposure [25]. Within a post-surgical context, there needs to be an adequate balance between pain control and over-prescribing. In a retrospective study of both open and MIS gynecologic oncology postoperative patients, it was found that 39% did not use opioids, and half of those who had a laparotomy used less than two opioid pills after discharge [26]. Interestingly, this study also demonstrated that opioid use was not impacted by open or MIS surgery [26]. A tiered opioid prescribing protocol in gynecologic oncology has been described, whereby differing prescription guidelines for differing surgical procedures and pain history are employed. In the studies described in our review, the majority of patients undergoing MIS gynecologic oncology procedures would be categorized as tier 2 and would receive five 5 mg oxycodone pills (or equivalent) at discharge, with patient satisfaction and pain scores not impaired by this approach [27]. This further raises the possibility of a time in the future when opioids are not routinely prescribed for home use after MIS procedures, although this is controversial. 

Finally, with regard to patient satisfaction, this remains an area of great importance that has not been adequately explored. Many of the studies included in our review reported balancing measures of patient satisfaction with regard to outcomes of interest, such as SDD, or pain scores. Perioperative patient satisfaction was quantified by Ferraioli et al. who found high general satisfaction rates following ERAS MIS gynecologic oncology surgery [16]. There remain areas of improvement in this realm that require novel thinking and study design, such as in a recent randomized clinical trial that implemented smartphone-assisted monitoring in the postoperative period in gynecologic oncology surgery following an ERAS protocol, 40% of whom have MIS surgery. This study found that the app-assisted follow-up post-discharge was associated with improved Quality of Recovery-15 scores and equal patient satisfaction [28]. Such interventions could also lead to reduced health resource requirements postoperatively, and further cost savings. 

## 5. Conclusions

In conclusion, ERAS in minimally invasive gynecologic oncology surgery is associated with significant positive impacts, specifically increased rates of SDD, decreased opioid use, improved satisfaction, and cost savings. ERAS MIS holds great promise for both patients and the healthcare system. 

## Figures and Tables

**Table 1:** Original research articles addressing benefits of ERAS in the context of MIS gynecologic oncology surgery.

Author (Year)	Pertinent Outcomes	Research Question	Method/Population	Results
Brancazio et al. (2021) [9]	Decreased length of stay	To identify factors to improve SDD in MIS GO surgery	Retrospective pre/post case–control study of ERAS MIS (10 months prior and 10 months after ERAS implementation)	*N* = 800: 351 ERAS MIS, 449 historical controls Use of a standardized ERAS protocol increased SDD (48.9% vs. 39.3%)
Lehman et al. (2021) [10]	Decreased opioid use	Impact of ERAS MIS in GO surgery on perioperative use of opioids	Used morphine mg IV equivalents	Opioid use less in ERAS MIS: 28.5 mg IV Eq vs. 23.6 mg IV Eq, *p* < 0.001) Nonsignificant trend to less opioids in recovery room (4.8 mg IV Eq vs. 4.1 mg IV Eq, *p* = 0.08)
Levytska et al. (2022) [11]		ERAS in MIS GO patients with respect to opioid use perioperatively	Used morphine milligram equivalents (MME)	Postoperatively in ERAS MIS: less opioids prescribed (197.8 vs. 223.5 MME, *p* = 0.0087) ERAS MIS cohort: LESS postoperative phone calls, calls for pain, and unscheduled visits due to pain
Wield et al. (2022) [12]	Decreased length of stay	To determine safety and feasibility of SDD after MIS GO surgery	Retrospective cohort study	*N* = 1124 69% SDD Predictors of admission: Demographics: older age, distance from hospital, Operative: longer procedure, complications, later start time, radical hysterectomy, adhesiolysis, mini-laparotomy Comorbidities: CAD, CVD, VTE, DM, neurologic disorders Rare adverse outcomes, with fewer overall complications in SDD
Chapman et al. (2016) [13]	Decreased length of stay Decreased opioid use Cost savings	To determine benefits of ERAS in MIS GO population	Retrospective case–control study of 2:1 ERAS MIS: historical matched controls	*N* = 165: 55 ERAS MIS 110 historical controls ERAS MIS: more likely to be discharged POD1 (91% vs. 60%, *p* < 0.001) median hospital stay 4 h less decreased postoperative pain (2.6 vs. 3.12, *p* = 0.03) 30% less opioid use Decreased hospital costs (USD 1810 per patient better (12%) *p* = 0.01)
Fernandez et al. (2023) [14]	Decreased length of stay	To compare length of stay after ERAS implementation in GO surgery	Retrospective pre/post case–control study of ERAS and historical matched controls (Included both laparotomy and MIS GO surgery)	Total cohort: *N* = 187: 103 ERAS, 84 historical controls ERAS MIS: *n* = 86 ERAS MIS and 65 historical controls Length of stay in ERAS MIS: 0.9 vs. 3.2 days (*p* < 0.0001)
Mateshaytis et al. (2022) [15]	Decreased length of stay	QI initiative to increase rate of SDD in endometrial cancer MIS surgery	Time series design pre/post-intervention with processing measures and balancing measures	Rate of same-day discharge increased to 78.3% (from 29.4% at baseline) after bundled ERAS MIS QI intervention
Ferraioli et al. (2019) [16]	Patient satisfaction	To evaluate patient satisfaction with ERAS program in MIS GO surgery	Observational retrospective study using EVAN-G validated questionnaire for perioperative patient satisfaction	N = 92 General satisfaction: 81.9 (range 41.6–100), with 60.8% very satisfied and 32.6% quite satisfied with the quality of care
Kim et al. (2022) [17]	Decreased length of stay	QI program to improve rates of SDD after MIS GO surgery	Pre- and post-intervention study comparing demographic and perioperative outcomes	*N* = 102 ERAS MIS, *n* = 100 historical controls SDD rates improved from 29% pre-intervention to 75% post-intervention (*p* > 0.001)
Mitric et al. (2023) [18]	Cost savings	To look at the economic impact of SDD in ERAS MIS GO surgery		*N* = 96 ERAS MIS, *n* = 101 historical controls Median total cost reduction per patient was CAD 1129 (CAD 7252 post-intervention vs. CAD 8381 pre-intervention)
Kim et al. (2021) [19]	Decreased opioid use	To evaluate a restrictive opioid prescription protocol	Median morphine milligram equivalents Used for comparison	Decreased median morphine milligram equivalents prescribed from 50 to 25 (*p* < 0.001). 54% used no opioids postoperatively No additional opioid refill requests
Lambaudie et al. (2020) [20]	Decreased length of stay	To create a nomogram for preoperative assessment of those who may benefit from early discharge	Prospective observational study of patients with GO surgery and an ERAS program	*N* = 230, 83.9% were treated with MIS ERAs MIS was an independent factor for early discharge within ERAS (OR 0.02, 95% CI 0–0.07, *p* < 0.001)
Modesitt et al. (2016) [21]	Decreased opioid use	To evaluate surgical outcomes before and after ERAS for major gynecologic surgery	Retrospective pre/post study of clinical outcomes, costs, patient satisfaction Full ERAS pathway for open procedures, “light” ERAS pathway for MIS	ERAS “light” protocol (included GO and non-GO MIS cases): *n* = 249 ERAS MIS and *n* = 324 historical controls Decreased opioid use Intraoperative: 0 vs. 13 mg, (*p* < 0.001) Postoperative: 15.0 vs. 23.6 mg (*p* < 0.001)
Weston et al. (2020) [22]	Decreased opioid use	To evaluate ERAS MIS GO surgery on opioid requirements and postoperative pain scores	Retrospective pre/post-ERAS MIS implementation study Used oral morphine equivalents (OME)	*N* = 127 in the ERAS MIS, *n*= 99 historical controls Adjusted for confounders, opioids in ERAS MIS cohort: Intraoperative 10.43 OME fewer (*p* < 0.001) Postoperative 10.97 OME fewer (*p* = 0.019) Pain scores: 0.56 lower in ERAS MIS cohort (*p* = 0.013)

ERAS: enhanced recovery after surgery; MIS: minimally invasive surgery; SDD: same-day discharge; GO: gynecologic oncology; QI: quality improvement; POD: post-op day; CAD: coronary artery disease; CVD: Cerebrovascular disease; VTE: venous thromboembolism; DM: diabetes mellitus.
